# One-Step Synthesis of W/O and O/W Emulsifiers in the Presence of Surface Active Agents

**DOI:** 10.1007/s11743-012-1404-6

**Published:** 2012-09-25

**Authors:** Elwira Sadecka, Halina Szeląg

**Affiliations:** Department of Fats and Detergents Technology, Chemical Faculty, Gdansk University of Technology, Narutowicza 11/12, 80-233 Gdansk, Poland

**Keywords:** Microemulsions, Esterification, Modified acyl propylene glycol emulsifiers, W/O and O/W emulsions

## Abstract

The main goal of this study was to describe the method of the synthesis of the dodecyl-, tetradecyl-, hexadecyl- and octadecyl-propylene glycol emulsifiers in the presence of selected anionic and nonionic surfactants. Acyl propylene glycol emulsifiers were produced by esterification of propane–1,2-diol (propylene glycol, PG) with C_12:0_–C_18:0_ fatty acids in the presence of anionic sodium dodecyl sulfate (SDS) and nonionic-poly(ethylene glycol) monolaurate (PEGML). The presence of SDS and PEGML in the reaction system caused microemulsion formation. Depending on the structure and amount of the surfactant in the system reactions proceeded at different rates and with different efficiency levels. The esterification of propylene glycol carried out under applied conditions causes products with the desired contents of propylene glycol monoesters (MAPG) to be obtained in a one-step reaction. Knowledge of the reaction kinetics creates the possibility to program the composition and properties of the synthesized emulsifiers. The interaction of nonionic, lipophilic MAPG with anionic, hydrophilic SDS or nonionic, hydrophilic PEGML influences the hydrophile–lipophile balance (HLB) values of the products which may be used to stabilize water-in-oil (W/O) and oil-in-water (O/W) emulsions. Use of the synthesized compounds allows stable emulsions to be prepared which include the following vegetable fats in the oil phase: mango oil, palm oil, shorea butter and hydrogenated soybean oil.

## Introduction

The ester derivatives of polyhydric alcohols and fatty acids belong to the group of nonionic surface active agents which are commonly used in the pharmaceutical, cosmetic and food industries as water-in-oil (W/O) emulsion stabilizers [1–5]. To the discussed group of compounds belong derivatives of propane–1,2-diol (propylene glycol, PG) and fatty acids (FA), of which the monoacyl propylene glycols (MAPG) play the most significant role. In most cases MAPG are the main components of acyl propylene glycol emulsifiers.

The commercial methods to obtain monoacyl propylene glycols are transesterification of triacylglycerols with propylene glycol and direct esterification of propylene glycol with fatty acids. The products of direct esterification are mixtures of mono- and diacyl propylene glycols and some unreacted substrates. Direct esterification is often carried out in the presence of catalysts, such as sodium hydroxide or lime (calcium hydroxide) [2] and at high temperatures (170–250 °C). Transesterification leads to the product that is a mixture of mono- and diacyl propylene glycols, mono-, di- and triacylglycerols and unreacted PG. The concentration of MAPG in products obtained with these methods falls in the range of 45–70 wt%. A higher concentration of monoesters can be obtained by molecular distillation of the product [6] or by extraction of the reaction mixture [7, 8].

Esterification of propylene glycol with a fatty acid can also be realized using enzymes as catalysts [9, 10]. Utilization of enzymes allows to realize the process of MAPG synthesis at lower temperatures and with a high conversion of the substrates (50–100 %).

One of the methods to obtain the ester derivatives of polyols (glycerol, propylene glycol, ethylene glycol) and fatty acids, is direct esterification of polyols with fatty acids realized in the presence of selected surfactants [11–14]. The proposed method of synthesis provides a few important advantages. The presence of surfactants allows a microemulsion to be formed. Formation of microdispersed system significantly increases the contact area between reagents and, as a consequence, reaction rate. Selection of an appropriate quantity and type of surfactant, type of fatty acids, temperature of the process and reaction time lead to obtain emulsifiers with the desired hydrophilic–lipophilic properties. This is particularly beneficial considering the use of the synthesized compounds as emulsifiers. Moreover, the discussed method of emulsifier synthesis is a one-step process occurring without harmful by-products and with materials from renewable sources used as reagents. The resultant products do not require purification and can be directly used as stabilizers in dispersed systems.

In our previous work, we focused on the possibility of obtaining dodecyl propylene glycol emulsifiers in the microemulsion system which is formed as a result of the insertion of anionic surfactant sodium dodecyl sulfate (SDS) to the reaction system. We presented the influence of the presence and concentration of sodium dodecyl sulfate on the esterification of propylene glycol with dodecanoic acid [14]. It was stated that the quantity of SDS significantly influenced reaction rate and concentration of MAPG in the product. Depending on the sodium dodecyl sulfate content in the reaction mixture, emulsifiers were obtained with HLB values within the range of 3.0–5.0.

This study has investigated the influence of fatty acid chain length and surfactant type on the esterification of propylene glycol with fatty acids (C_12:0_–C_18:0_) in the presence of sodium dodecyl sulfate (SDS) and poly(ethylene glycol) monolaurate (PEGML). For characterization of MAPG formation kinetic studies were examined. The dependencies of the hydrophilic–lipophilic properties (HLB) of the synthesized products on fatty acids structure were also studied. Emulsions stabilized with the synthesized emulsifiers were prepared. The influence of emulsifier type and concentration and the type of the oil phase on the stability of the obtained dispersions was also investigated. As the oil phase vegetable fats, e.g., palm oil, soybean oil, mango oil and shorea butter, were used.

## Experimental

### Materials

The fatty acids (FA): dodecanoic (lauric, C_12:0_, 98.8 wt%), tetradecanoic (myristic, C_14:0_, 98.9 wt%), hexadecanoic (palmitic, C_16:0_, 91.5 wt%) and octadecanoic (stearic, C_18:0_, 92.2 wt%), were purchased from Aldrich-Chemie GmbH & Co. KG, Germany. Propane–1,2-diol (propylene glycol, PG, 99.7 wt%) was obtained from PPH POCh, Poland. Sodium dodecyl sulfate (99 wt%) was obtained from Alfa Aesar GmbH & Co KG, Germany. Polyoxyethylated ethylene glycol monolaurate (PEG) was obtained from Croda, Great Britain.

To prepare model emulsions a mixture of paraffin oil (Shell Ondina 934, medical purity) and paraffin wax (Sigma-Aldrich) (9:1 w/w) was used as the oil phase.

Also prepared were emulsions including in the oil phase the following vegetable fats: (AarhusKarlshamn, Sweden):

- Shorea butter (Lipex 106)—vegetable fat from the fruit of *Shorea stenoptera*.
- Soybean oil (Akofine S)—hydrogenated, solid soybean oil.
- Palm oil (Lipex Genova)—semi-liquid emollient derived from palm oil.
- Mango oil (Lipex 203)—fat derived from mango fruit (*Magnifera indica*).

The compositions of the fatty acids of the fat components used are presented in Table 1.

**Table 1:** The compositions of the fatty acids of the oil components used

Fatty acid	Shorea butter	Hydrogenated soybean oil	Mango oil	Palm oil
<C_14:0_	–	0.1	–	0.2
C_14:0_	0.1	0.1	0.1	1.0
C_16:0_	19.5	11.4	10.5	43.9
C_16:1_	0.1	–	0.2	0.1
C_16:2_	0.2	–	–	0.1
C_18:0_	43.9	86.1	27.5	4.5
C_18:1_	32.9	1.1	51.8	39.3
C_18:2_	0.9	0.1	5.6	9.8
C_18:3_	0.1	–	0.5	0.2
C_20:0_	2.1	0.5	2.4	0.4
C_20:1_	–	–	0.4	0.1
C_22:0_	0.1	0.3	0.5	0.1

### Esterification of Propylene Glycol with Fatty Acids

Reactions were conducted in a thermostatic reactor equipped with a stirrer, a thermometer and a nitrogen tube. The stirring rate was adjusted to 200 rpm. Esterifications were carried out under reduced pressure (800 hPa) to eliminate water that was formed during the esterification process. All syntheses were carried out at a temperature of 150 (±1 °C) for 6 h.

Propylene glycol was added to the heated mixture of proper fatty acid and one of the surface active agents (SDS or PEGML) used. The molar ratio of fatty acid to propylene glycol—1:1.25—was constant in all of the esterification processes. The concentration of sodium dodecyl sulfate was 0.01 mol and nonionic surfactant 0.05. This concentration of the surfactants in the reaction mixture was experimentally chosen taking into consideration possibility to obtain products with maximal MAPG content.

The progress of the esterification reaction was investigated by analyzing the composition of the reaction mixture at 1-h intervals. The estimation of the standard error of the fatty acids, propylene glycol and MAPG concentration analyses from three separate reactions conducted under the same conditions revealed maximum confidence intervals of ±0.7 % for FA and MAPG and ±1.1 % for PG.

### Analytical Methods

The concentrations of PG and MAPG in the products were determined as trimethylsilyl derivatives by programmed GC with internal standardization [11]. The FA concentrations were determined by the potentiometric titration method, according to the IUPAC method [15]. The total amount of diesters of propylene glycol (DAPG) was calculated taking into account the concentrations of the other compounds in the reaction mixture. For statistical evaluation of the analytical methods, the concentration of fatty acids, propylene glycol and MAPG were checked five times in a few samples of the reaction mixture. The maximum confidence intervals were ±0.7 % for FA and ±0.9 % for MAPG and PG.

### Hydrophile–Lipophile Balance (HLB) of the Emulsifiers

The HLB values were estimated experimentally by the Griffin method [16]. The composition of the emulsion during the evaluation of HLB was as follows: oil phase (paraffin oil/paraffin wax, 9:1 w/w): 40 wt%; water: 55 wt%; emulsifier: 5 wt%. As a standard Tween 60 (HLB = 14.9) and Span 60 (HLB 4.7) emulsifiers were used. The confidence interval of the HLB values was 0.1.

### Preparation of the Emulsions

The model emulsions were prepared with the following oil to water phase weight ratios: (O/W): 20:80; 30:70; 40:60; 50:50; 60:40; 70:30; 80:20. The emulsifier constituted 10 wt% of the emulsion. As the oil phases, a mixture of the paraffin oil and paraffin wax (9:1 w/w) was used. Emulsions were also prepared which included paraffin oil, paraffin wax and vegetable fats (mango oil, palm oil, shorea butter and soybean oil) in the oil phase.

### The Stability of the Emulsions

The stability of the prepared emulsions was measured by the DLS (Dynamic Light Scattering) method using Turbiscan TLab Expert (Formulaction, France). The dispersions were placed in a flat-bottomed cylindrical glass cell. The sample was scanned by using two synchronous optical sensors that detected light transmitted through the sample. The reading head acquired the transmission data every 40 μm while moving along the entire height of the cell. The light source was an electro-luminescent diode. The scanning was carried out at room temperature.

## Results and Discussion

### Characterization of the Esterification Process

To check the influence of surfactant structure and fatty acid hydrocarbon chain length on the reaction progress and the properties of the obtained emulsifiers the esterifications of PG with fatty acids (C_12:0_–C_18:0_) were conducted according to the method described above.

In all the esterification processes, the formation of the microemulsion was observed at the reaction temperature. The system became transparent just after the beginning of the reaction.

During the studies, the influence of fatty acid type and the type and concentration of the surfactant on the reaction progress, the amount of PG monoesters and the values of reaction rates were examined.

Taking the analytical data into consideration, the conversion of fatty acid (α_FA_) and propylene glycol (α_PG_) was determined (Eq. 1):

1 $$ \alpha_{FA} = \frac{{(FA_{0} - FA)}}{{FA_{0} }}\quad \alpha_{PG} = \frac{{(PG_{0} - PG)}}{{PG_{0} }} $$

FA and PG represent fatty acid and propylene glycol concentrations (wt%) in real time in the reaction mixture, FA_0_ and PG_0_ represent concentrations (wt%) of proper fatty acid and propylene glycol at the beginning of the reaction.

**Fig. 1:**
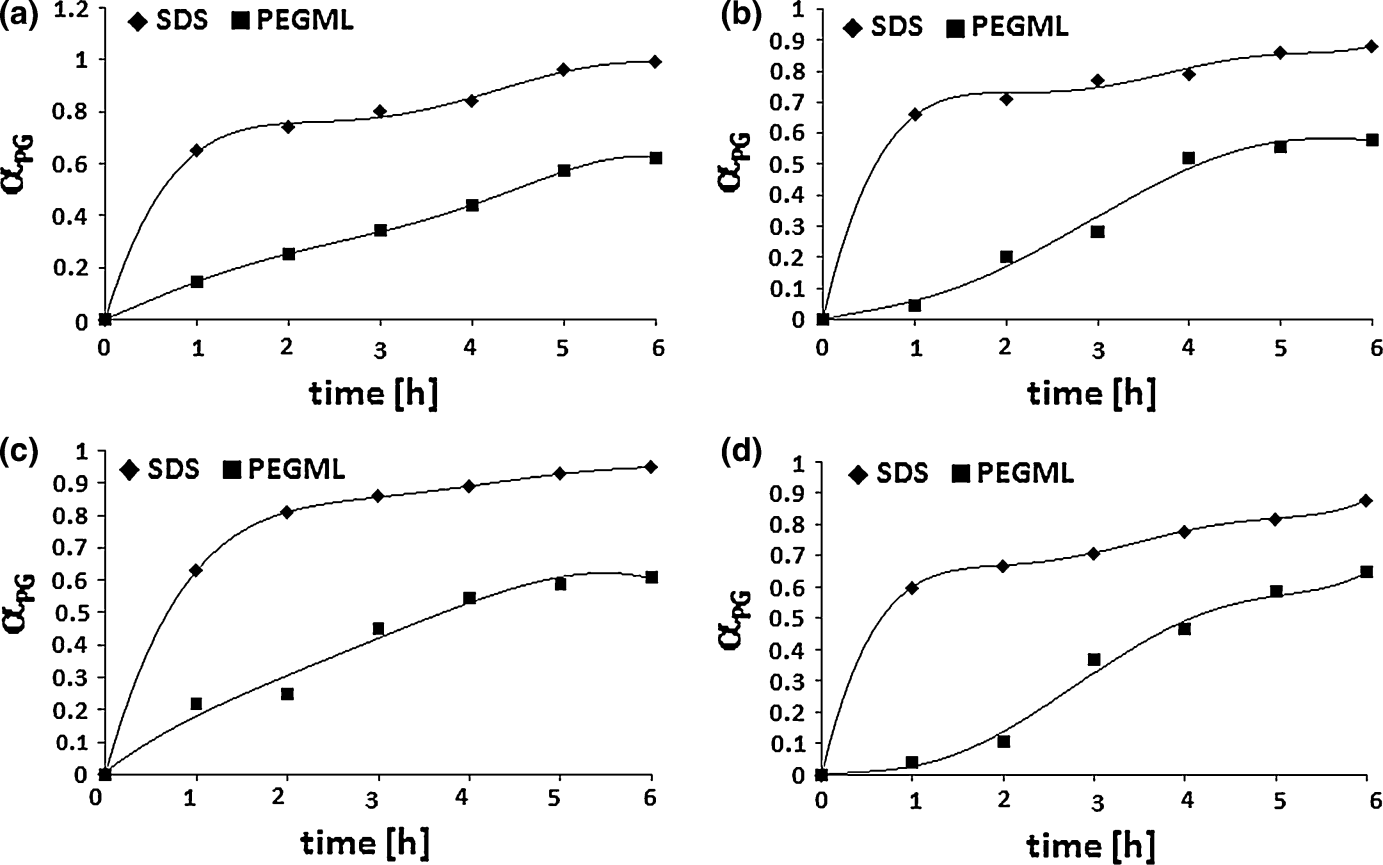
The influence of fatty acids structure on propylene glycol conversion (α_PG_) during esterification of PG with fatty acids in the presence of SDS and PEGML. The molar ratio of the reagents: PG/FA/SDS (PEGML) 1.25:1.0:0.01 (0.05). FA: C_12:0_ (**a**); C_14:0_ (**b**); C_16:0_ (**c**); C_18:0_ (**d**)

Because of the different molar ratios of the SDS and PEGML used in the reaction mixtures, the comparison the influence of the fatty acid structure depending on surfactant type and the concentration on the reaction progress is quite difficult. Nevertheless, it was observed that in the case of reactions carried out in the presence of 0.01 mol of SDS, i.e. at a concentration five times lower than the concentration of PEGML, the conversion of propylene glycol (α_PG_) as well as the conversion of fatty acids (α_FA_) were much higher regardless of the structure of the fatty acid used in the synthesis. For example, when reactions were conducted in the presence of SDS the smallest α_PG_ value was 0.88 (using octadecanoic fatty acid) and the highest 0.99 (when C_12:0_ and C_14:0_ fatty acids were used) after 6 h of the process. When the reaction was carried out in the presence of PEGML the maximal α_PG_ values fell in the range of 0.58 ÷ 0.65 depending on fatty acid structure and were obtained after 6 h from the beginning of the reaction (Fig. 1).

**Fig. 2:**
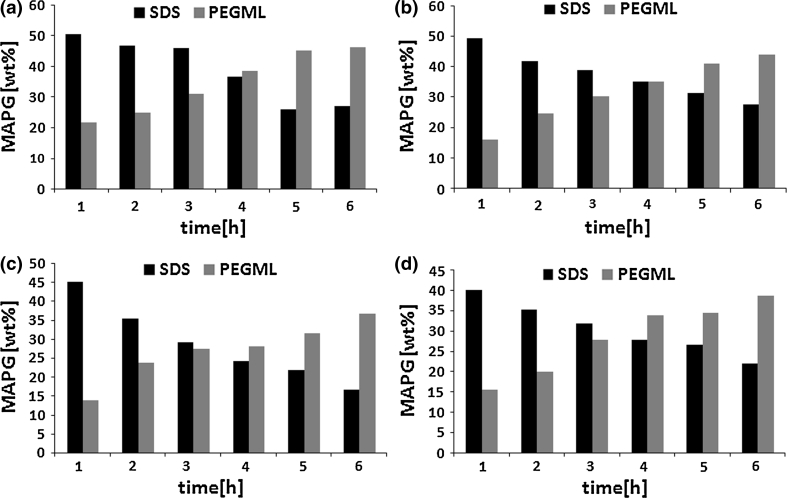
The influence of fatty acids structure on concentration of MAPG in the reaction mixture. The molar ratio of the reagents: PG/FA/SDS (PEGML) 1.25:1.0:0.01 (0.05). FA: C_12:0_ (**a**); C_14:0_ (**b**); C_16:0_ (**c**); C_18:0_ (**d**)

Comparing the influence of fatty acid type on propylene glycol monoester concentration in the reaction mixture it was observed that increasing fatty acid chain length resulted in decreasing MAPG content in the system (Fig. 2) for reactions conducted in the presence of SDS as well as using PEGML.

It is noteworthy that insertion into the system of 0.01 mol of anionic surfactant led to products including maximally from 40.1 to 50.5 wt% MAPG, depending on the fatty acid structure. When PEGML was used in the process the maximal concentration of PG monoesters was in the range of 36.6–46.2 wt%. However, to obtain products with such a content of MAPG the amount of PEGML should be five times larger than that of SDS and the time needed to obtain products with a maximal concentration of MAPG was significantly higher.

Based on the results of particle size analysis of the products synthesized in the esterification of PG with dodecanoic acid in the presence of SDS [14] we showed, that the presence of this surface active agent in the reaction system leads to the microemulsion formation. We suppose SDS works as an emulsifying agent at the beginning of the process which results in increasing the PG/dodecanoic acid interface. During the esterification of PG with dodecanoic acid a mixed SDS/MAPG film forms and decreases the interfacial tension between phases to a value which permits the formation of microemulsion. A similar phenomenon can be observed when esterification of PG is realized using the other fatty acids and in the presence of PEGML. However, in the case of the use of dodecanoic acid, formation of the microemulsion resulting from the interaction between surfactants revealed structural similarity: dodecyl propylene glycol and sodium dodecyl sulfate (or PEGML). Thus, it is possible that this mixed surfactant film is more effective in decreasing the interfacial area between fatty acid and PG in comparison to mixed surfactant films which are formed using other fatty acids.

### Reaction Rate Constants

During the esterification of propylene glycol with fatty acids, water is formed, which could result in the hydrolysis of acyl propylene glycol. Under the conditions applied in the processes discussed, water was removed from the reaction environment, so this disadvantageous reaction could be avoided. Thus, the process can be characterized as an irreversible reaction. The appropriate fatty acid reacts with propylene glycol, forming MAPG which is esterified to propylene glycol diester (DAPG) in the next step of the reaction.

Taking into account the concentration changes of PG, FA, MAPG and DAPG during the process, the esterification of propylene glycol with C_12:0_–C_18:0_ fatty acids in the presence of SDS and PEGML can be described as a consecutive reaction and MAPG is an intermediate product.

The reaction kinetic can be described with the following expression with respect to propylene glycol esterification (Eq. 2):

2 $$ \begin{gathered}
{\text{PG}}\mathop{\longrightarrow}\limits^{{k_{1}
}}{\text{MAPG}}\mathop{\longrightarrow}\limits^{{k_{2}
}}{\text{DAPG}} \hfill \\ \hfill \\ \end{gathered} $$

Taking into consideration the consecutive character of the reaction, we described the esterification of propylene glycol with C_12:0_–C_18:0_ fatty acids in the presence of SDS and PEGML using equations included in our previous works [11–14]. Based on these equations the rate constants of MAPG (*k*_1_) and DAPG (*k*_2_) formation, the maximum concentration of monoesters (MAPG_max_) and the time when such a concentration of MAPG can be reached (*t*_max_) were calculated.

Comparison of the progress of esterification of PG with fatty acids in the presence of SDS and PEGML leads to the conclusion that the structure of fatty acids influenced rate constants of MAPG (*k*_1_) and DAPG (*k*_2_) formation, as well as the values of MAPG_max_ (Table 2) in both cases.

**Table 2:** The rate constants of esterification of propylene glycol with fatty acids C_12:0_–C_18:0_ in the presence of SDS and PEGML

Reaction	k_1_ × 10^5^ [s^−1^]	k_2_ × 10 [s^−1^]	MAPG_max_	*t*_max_
PG:FA:SDS				
1.0:1.25:0.01				
PG:C_12:0_:SDS	19.9	9.8	54.3	1.9
PG:C_14:0_:SDS	14.3	11.9	44.4	2.0
PG:C_16:0_:SDS	15.8	12.6	45.4	2.0
PG:C_18:0_:SDS	13.9	14.1	40.9	2.0
PG:FA:PEGML				
1.0:1.25:0.05				
PG:C_12:0_:PEG	5.2	3.4	44.4	6.5
PG:C_14:0_:PEG	4.3	3.7	41.4	6.9
PG:C_16:0_:PEG	4.5	4.2	40.4	6.3
PG:C_18:0_:PEG	4.8	4.1	42.9	6.2

The molar ratios of the substrates: PG:FA:SDS 1.25:1.0:0.01 and PG:FA:PEGML 1.25:1.0:0.05

Analyzing the values of rate constants of esterification realized using SDS it was observed that *k*_1_ values decreased with an increase in the fatty acid chain length. At the same time *k*_2_ values increased. According to the above, the amount of the MAPG in the products also decreased. As can be seen from Table 2, the compound with the highest PG monoester concentration (54.3 wt%) could be synthesized when propylene glycol was esterified with dodecanoic acid. Use of tetra-, hexa- and octadecanoic fatty acids resulted in a decrease in the *k*_1_/*k*_2_ ratio and, as a consequence, in a decrease in the MAPG concentration in the products. Regardless of fatty acid type, the time needed to obtain products with the highest MAPG amount did not change and was about 2 h.

In the case of esterification of PG which occurred in the presence of PEGML the highest rate constant value of MAPG formation (*k*_1_) was also obtained in the reaction of PG with dodecanoic acid (Table 2). The growth of the fatty acid chain length (to C_14:0_) caused a decrease in the *k*_1_ value. However, a further increase in the number of carbon atoms in the fatty acid molecule resulted in the *k*_1_ value increasing with a simultaneous increase in the *k*_2_ rate constant value. The decrease in the *k*_1_/*k*_2_ ratio for the reaction realized with C_12:0_; C_14:0_ and C_16:0_ fatty acids caused a decrease in the MAPG_max_ value. The maximal concentrations of PG monoesters were 44.4 wt% for dodecanoic acid, 41.4 and 40.4 wt% for tetra- and hexadecanoic acids respectively. When octadecanoic fatty acid was used, the *k*_1_/*k*_2_ ratio increased and the maximal concentration of propylene glycol monoesters in the product also increased (to 42.9 wt%).

### Hydrophile–Lipophile Balance (HLB)

Based on the kinetic data we synthesized emulsifiers with about 35.5–50.2 wt% MAPG concentration. The values of the hydrophile–lipophile balance of the obtained products (Table 3) and required HLB values of the used oil phases were determined according to the Griffin method [16].

**Table 3:** The HLB values of the emulsifiers obtained in esterification of PG with fatty acids (C_12:0_–C_18:0_) in the presence of sodium dodecyl sulfate (SDS) and poly(ethylene glycol) monolaurate (PEGML)

Emulsifier*	MAPG (wt%)	SURFACTANT (wt%)	HLB
PG:C_12:0_:SDS	50.2	0.9	5.0
PG:C_14:0_:SDS	45.2	0.9	3.4
PG:C_16:0_:SDS	46.0	0.9	5.6
PG:C_18:0_:SDS	41.4	0.9	5.8
PG:C_12:0_:PEGML	35.7	6.1	26–40
PG:C_14:0_:PEGML	35.8	5.5	12–40
PG:C_16:0_:PEGML	35.5	5.2	10–40
PG:C_18:0_:PEGML	38.0	4.8	7–40

*The molar ratio of the reagents: PG:FA:SDS 1.25:1.0:0.01 and PG:FA:PEGML 1.25:1.0:0.05 T = 150 °C

The HLB values of the dodecyl-, tetradecyl-, hexadecyl and octadecyl propylene glycol emulsifiers modified with SDS are characterized by hydrophobic properties and can be used as W/O emulsion stabilizers (Table 3). The compounds synthesized in the presence of PEGML demonstrate hydrophilic properties—their HLB values are within the range of 7–40, depending on the fatty acid structure. The HLB values obtained show that products obtained in the presence of PEGML can be used as O/W emulsion type stabilizers.

Based on the generated data we can conclude that depending on the fatty acid structure and surfactant type, emulsifiers can be synthesized with HLB values over a relatively wide range. It creates the possibility of programing the esterification in such a way as to allow products with desired hydrophile–lipophile properties to be obtained, i.e. with HLB values which precisely suit the required HLB value (RHLB) of the oil phase which we want to use in the emulsion. Emulsifier HLB values with high levels of suitability produce the most stable emulsions in the long run [17].

It is worth noting that the presence of SDS in the system significantly influenced the reaction progress, but only insignificantly influenced the hydrophile–lipophile properties of the products. In the case of the use PEGML in the process, PEGML presence was not observed to have a substantial influence on the reaction rate. However, the presence of nonionic surfactant in the emulsifier composition drastically changed the hydrophile–lipophile properties of acyl propylene glycol emulsifiers, which reveal hydrophobic properties after all. Such a different influence as a consequence of the presence of surfactant on the esterification progress and the HLB value of MAPG emulsifiers may be a result of the nature of PEGML and SDS and their behavior at the water/oil interface. PEGML is usually used as an effective O/W emulsifier (HLB = 13.9). SDS, due to its much stronger hydrophilic properties (HLB = 40), tends to dissolve in the hydrophilic phase of the dispersed system in contrast to PEGML which orientates at the interface and decreases the interfacial tension to produce a stable emulsion.

### Use of Acyl Propylene Glycol Emulsifiers Modified with Surfactants to Stabilize Water-In-Oil and Oil-In-Water Emulsions

One of the most important parts of the experiments was to define the possibility of using the synthesized emulsifiers to stabilize the emulsion systems.

In the first step of the research model, emulsions were prepared stabilized with SDS–modified emulsifiers and including a mixture of paraffin oil and paraffin wax in the oil phase. The compositions of the compounds applied are presented in Table 3. Based on the stability of the obtained emulsions the optimal composition of the oil to water phase was specified. It was shown that the most stable emulsions could be prepared when the water to oil phase weight ratios (W/O) were 40:60 and 30:70.

For the preparation of the next emulsions mixtures of paraffin oil, paraffin wax and vegetable fats (shorea butter, mango oil, soybean oil and palm oil) in different weight ratios were used as the oil phases. The W/O weight ratios were 40:60 and 30:70. Such weight ratios were chosen according to the stability of the model emulsions.

The stability of the prepared emulsion systems was determined by measurements of the intensity (%) of backscattering light (BS) as a function of the storage time at room temperature using the DLS method. It is assumed that good stability is characterized by emulsions with BS values higher than 50 % after a definite time of storage.

**Fig. 3:**
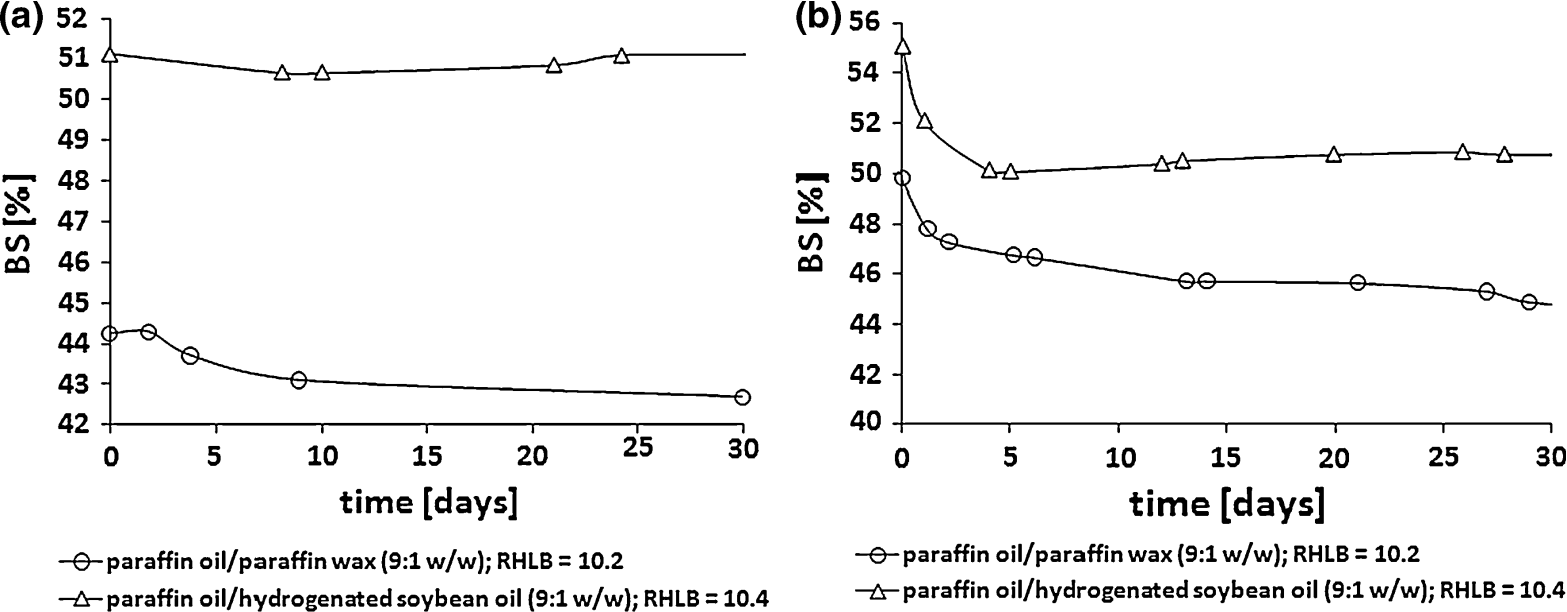
The influence of the type of the oil phase on the stability of the emulsions stabilized with acyl propylene glycol emulsifiers synthesized in the presence of SDS. W/O weight ratio 40:60. Emulsifier: PG:FA (C_16:0_ (**a**) and C_18:0_ (**b**)) :SDS 1.25:1.0:0.01

**Fig. 4:**
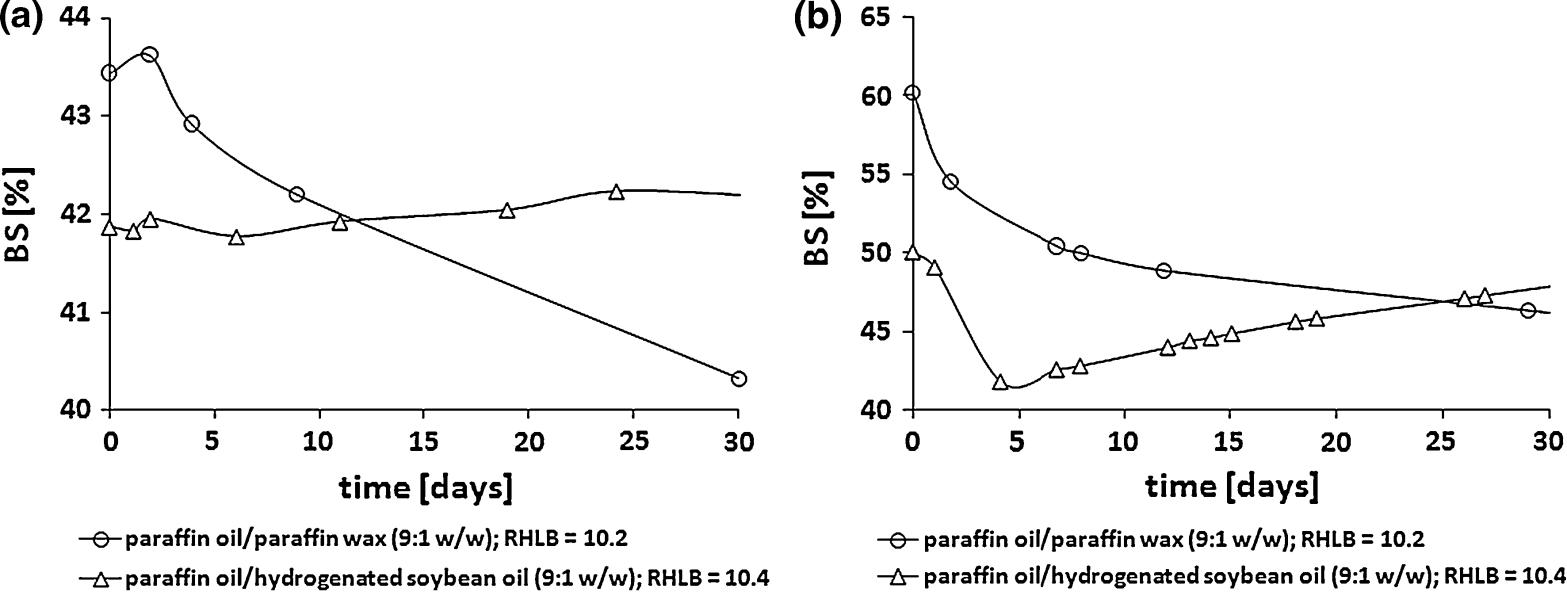
The influence of the type of the oil phase on the stability of the emulsions stabilized with acyl propylene glycol emulsifiers synthesized in the presence of SDS. W/O weight ratio 30:70. Emulsifier: PG:FA (C_16:0_ (**a**) and C_18:0_ (**b**)) :SDS 1.25:1.0:0.01

The experiments showed that stable W/O emulsions (W/O 40:60 and 30:70) could be prepared only with the emulsifiers synthesized in the reaction of PG with hexa- and octadecanoic acids, realized in the presence of SDS and when the hydrogenated soybean oil was used as a substitute for paraffin wax (Figs. 3, 4).

Insertion of palm oil or shorea butter into the oil phase instead of paraffin wax resulted in an intense decrease in emulsion stability or indeed total destabilization of the emulsion.

It was found that the degree of structural similarity between hydrocarbon fragments of the oil phase and the emulsifier plays a significant role in emulsion stability [18, 19]. In the discussed cases, stable emulsions were obtained only with the use of hexa- and octadecyl propylene glycol emulsifiers and when the oil phase of the emulsions was a mixture (9:1 w/w) of paraffin oil and hydrogenated soybean oil. Paraffin oil is a mixture of liquid alkanes, but soybean oil mainly contains palmitic (C_16:0_) and stearic (C_18:0_) fatty acids. Therefore, it may be assumed that the stability of the emulsions obtained results from both the structure of the emulsifiers used and their structural analogies with the oil phase.

As is commonly known, stable emulsions are best formulated with emulsifiers or a combination of emulsifiers with HLB (hydrophile–lipophile balance) values close to that required of the oil phase [17]. However, the HLB values of hexa- and octadecyl propylene glycol emulsifiers (5.6 and 5.8 respectively) are not compatible with the RHLB value of the oil phase used which is a mixture of paraffin oil and hydrogenated soybean oil (9:1 w/w, RHLB = 10.4). We may assume that it could result from the composition of the emulsifiers which are mixtures of components with different activities at the water/oil interface. According to the Griffin assumption, the HLB of the emulsifier mixture is not always equal to the required HLB value of the oil phase in stable emulsion systems, which can be the result of a different division of the oily products between the oil phase and the interphase [16].

**Fig. 5:**
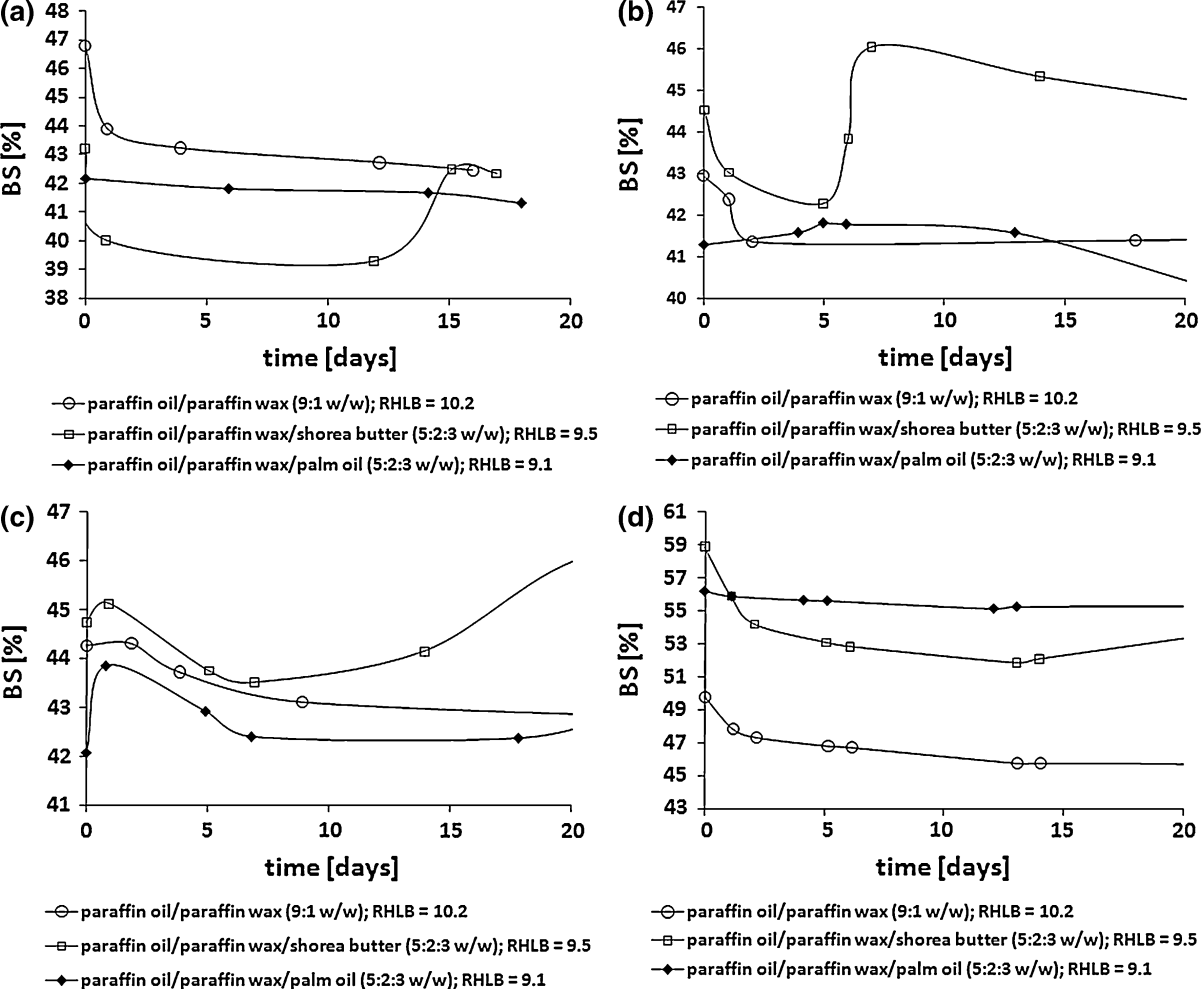
The influence of the oil phase on the stability of the emulsions stabilized with acyl propylene glycol emulsifiers synthesized with SDS. W/O weight ratio 40:60. Emulsifier: PG:FA (C_12:0_ (**a**); C_14:0_ (**b**), C_16:0_ (**c**) and C_18:0_ (**d**)) :SDS 1.25:1.0:0.01

**Fig. 6:**
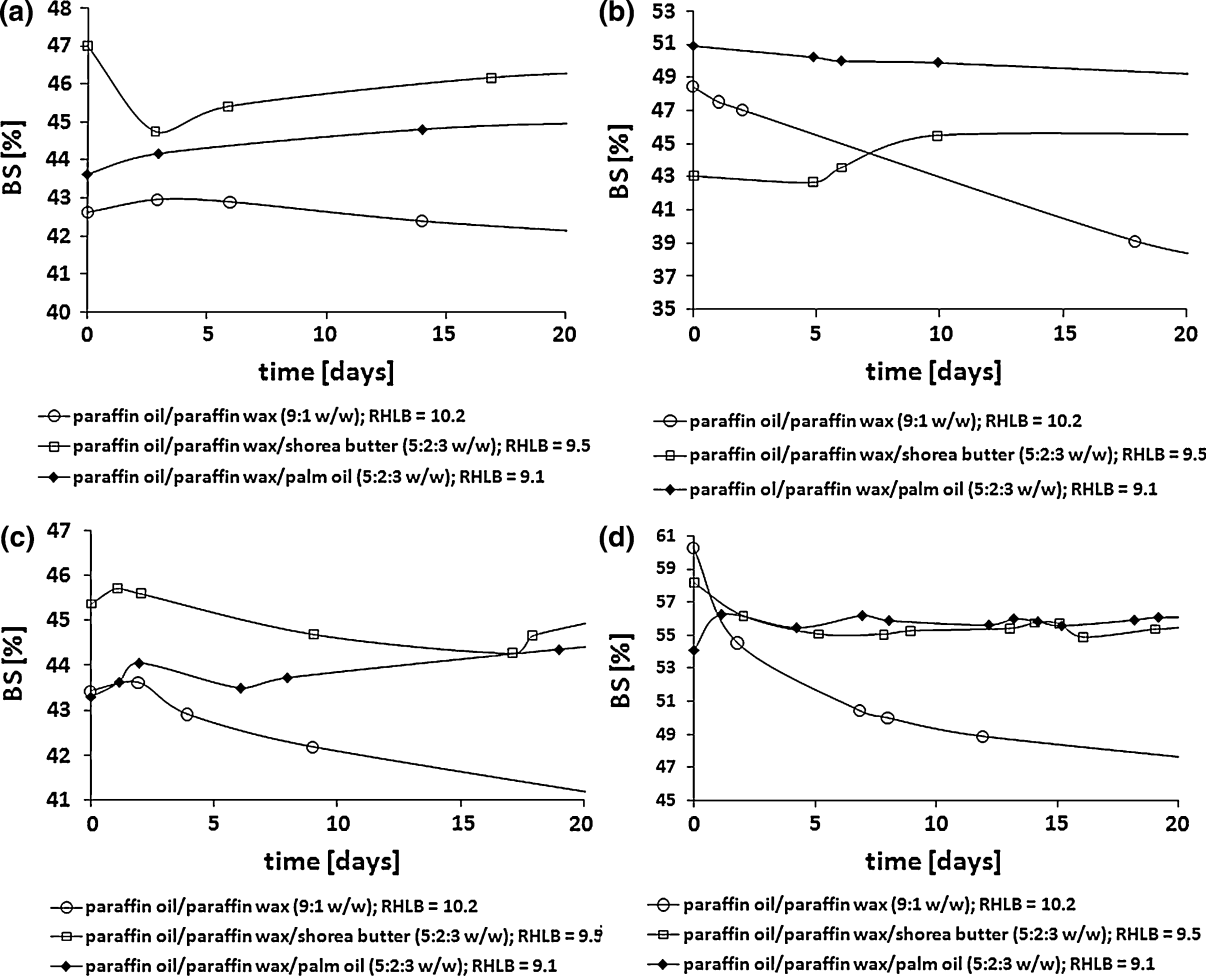
The influence of the oil phase on the stability of the emulsions stabilized with acyl propylene glycol emulsifiers synthesized with SDS. W/O weight ratio 30:70. Emulsifier: PG:FA (C_12:0_ (**a**); C_14:0_ (**b**), C_16:0_ (**c**) and C_18:0_ (**d**)) :SDS 1.25:1.0:0.01

Using a mixture of paraffin oil, paraffin wax and palm oil or shorea butter in a 5:2:3 weight ratio as the oil phase led to emulsions (W/O 40:60 and 30:70) with BS values of 40 % for almost all cases being obtained after about 20 days of storage at room temperature (Figs. 5, 6).

In the cases discussed, the compatibility of the HLB values between the oil phase (paraffin oil/paraffin wax/palm oil or shorea butter 5:2:3 w/w, RHLB = 9.1 and 9.5 respectively) and SDS–modified emulsifiers was not observed, similar to the cases described above.

The next stage of the experiments concerned the investigation of the possibility of using the acyl propylene glycol emulsifiers modified with PEGML to stabilize O/W emulsions containing shorea butter, mango oil, hydrogenated soybean oil and palm oil in the oil phase.

To obtain the first series of emulsions, dodecyl propylene glycol emulsifier synthesized in the presence of 0.05 mol of PEGML was utilized. The experiments showed that this emulsifier was ineffective in the stabilization of model emulsions including paraffin oil and paraffin wax (9:1 w/w) in the oil phase. Therefore, it seems to be tempting to assume that the instability of the obtained emulsions was a result of either the low surface activity of emulsifiers at the water/oil interface or the type of the oil phase. To determine the influence of the oil phase type on the stability of the emulsions a mixture of paraffin oil/(paraffin wax/shorea butter) (1:1 w/w) was used. The weight ratios of paraffin wax and shorea butter were 70:30; 60:40; 50:50 and 40:60. The O/W weight ratios were: 80:20; 70:30; 60:40; 40:60; 30:70 and 20:80.

It was shown that a significant influence on emulsion stability was exerted by the oil to water weight ratio but not by the relationship between HLB//RHLB values. For emulsion systems containing 20, 40 and 60 wt% of water and stabilized with dodecyl propylene glycol emulsifier synthesized in the presence of 0.05 mol of PEGML (HLB = 26–40), total destabilization was observed either after a few days of storage at room temperature or just after the end of the emulsification. This may be a result of the huge difference between hydrophile–lipophile properties of the emulsifiers and oil phase. However, for the other systems (O/W 30:70 and 20:80) increasing of shorea butter amounts in the oil phase also increased the stability (measured by the intensity of BS) of the emulsions. The highest stability was characterized by emulsions containing 70 wt% of water (BS above 60 %) in which the oil phase was a mixture of paraffin oil and (paraffin wax/shorea butter 40:60 w/w, RHLB 9.5) in the weight ratio 1:1.

**Fig. 7:**
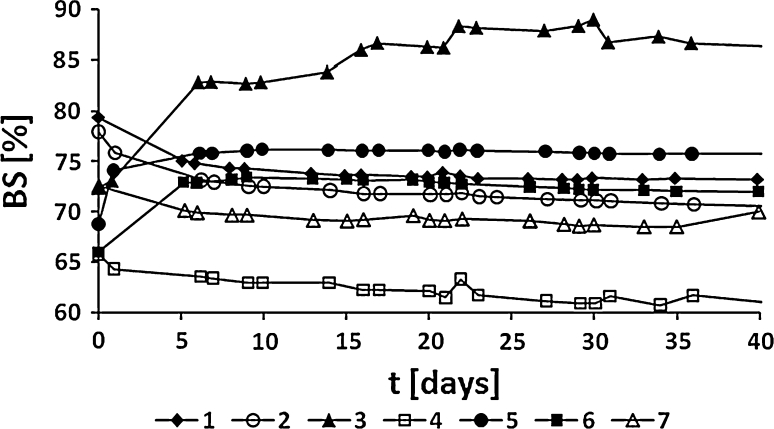
The dependence of the BS value [%] as a function of time (*t*) for the emulsions with 30:70 O/W ratio, varying the composition of the oil phase. Emulsifier: PG:FA:PEGML (1.25:1.0:0.005)/Span 60 1:1 w/w. Oil phase: paraffin oil/paraffin wax 9:1 w/w (RHLB = 10.2) (*1*); paraffin oil/shorea butter 9:1 w/w (RHLB = 10.5) (*2*); shorea butter (RHLB = 9.6) (*3*); mango oil (RHLB = 8.2) (*4*); palm oil (RHLB = 9.0) (*5*); mango oil/hydrogenated soybean oil 9:1 w/w (RHLB = 8.2) (*6*) and mango oil/shorea butter 1:1 w/w (RHLB = 7.3) (*7*)

**Fig. 8:**
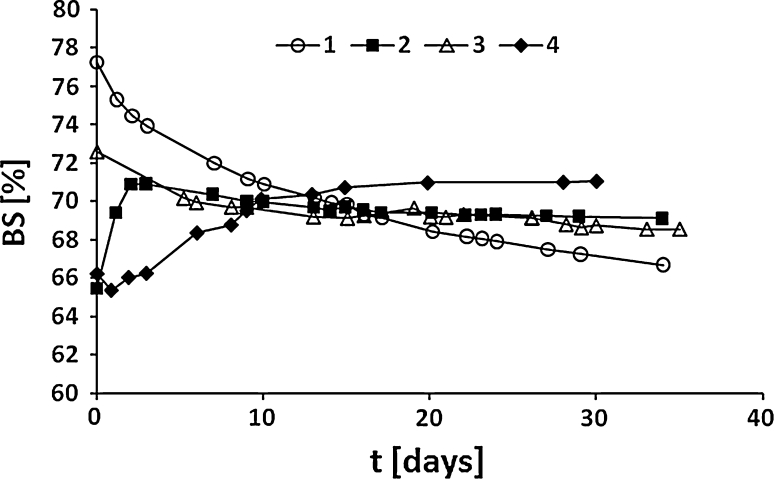
The dependence of BS value [%] as a function of time (*t*) for the emulsions with 20:80 O/W ratio, varying the composition of the oil phase. Emulsifier: PG:FA:PEGML (1.25:1.0:0.005)/Span 60 1:1 w/w. Oil phase: paraffin oil/shorea butter 9:1 w/w (RHLB = 10.5) (*1*); mango oil/hydrogenated soybean oil 9:1 w/w (RHLB = 8.2) (*2*); mango oil/shorea butter 1:1 w/w (RHLB = 8.9) (*3*) and mango oil/shorea butter/hydrogenated soybean oil 5:2:3 w/w (RHLB = 7.7) (*4*)

To obtain stable emulsions, a mixture of dodecyl propylene glycol emulsifiers synthesized in the presence of 0.005 mol of PEGML (HLB = 14–40) with sorbitan monostearate (Span 60, HLB = 4.7) in a 1:1 weight ratio was used as the emulsifier (HLB = 9.4–22.4). The composition of the PEGML-modified emulsifier was as follows: FA (C_12:0_): 23.0 wt%; PG: 16.4 wt%; MAPG: 42.4 wt%; DAPG: 17.2 wt% and PEGML 1.0 wt%. The oil to water weight ratios were 30:70 (Fig. 7) and 20:80 (Fig. 8). The oil phase of the prepared emulsions consisted of paraffin oil, paraffin wax and vegetable fats (Table 1).

As can be seen from Figs. 7, 8 the emulsions obtained are characterized by high stability (BS above 60 %) during storage at room temperature for about 40 days. High stability of the emulsions may be a result of the presence of synergetic operating emulsifiers in the system: hydrophobic MAPG, Span 60 and hydrophilic PEGML, in spite of the significant differences of hydrophilic–lipophilic properties between these compounds. The stability of the emulsions may also depend on the type of oil components used. An increase in BS value was observed for emulsions including hydrogenated soybean oil in the oil phase, which shows structural similarity to Span. For the other emulsion a decrease in the BS value over time was noticed. Neither of the emulsions analyzed underwent destabilization during storage at room temperature. The high stability of the emulsion may also be a result of the wide range of the HLB values of the mixture of emulsifiers used which are close to the RHLB values of the oil phases (from 7.3 to 10.2, depending on the oil phase type).
